# Supplementary material for: Prevalence of Avascular Necrosis Following Surgical Treatments in Unstable Slipped Capital Femoral Epiphysis (SCFE): A Systematic Review and Meta-Analysis
Source: Children (Basel). 2022 Sep 11;9(9):1374. doi: 10.3390/children9091374 (PMC9497816; doi:10.3390/children9091374)
Supplement: Supplementary file 1 [file children-09-01374-s001.zip › children-1885128-supplementary.pdf]

Supplementary Tables and Figures

Table S1. Search strategies.

Databases	Search strategies
PubMed	((((((((((((Epiphysiolysis Capitis Femoris[Title/Abstract]) OR Slipped Femoral Capital Epiphyses[Title/Abstract]) OR Slipped Capital Femoral Epiphysis[Title/Abstract]) OR Slipped upper femoral epiphysis[Title/Abstract]) OR Adolescent Coxa Vara[Title/Abstract]) OR Adolescent Coxa Varas[Title/Abstract]) OR Unilateral Slipped Capital Femoral Epiphyses[Title/Abstract]) OR Unilateral Slipped Capital Femoral Epiphysis[Title/Abstract]) OR Bilateral Slipped Capital Femoral Epiphyses[Title/Abstract]) OR Bilateral Slipped Capital Femoral Epiphysis[Title/Abstract]) OR SCFE[Title/Abstract]) OR Slipped capital epiphysis[Title/Abstract]) OR Unstable slip[Title/Abstract])) AND ((((((((Femur Head Necroses[Title/Abstract]) OR Femur Head Necrosis[Title/Abstract]) OR femoral head Necrosis[Title/Abstract]) OR femoral head osteonecrosis[Title/Abstract]) OR FHO[Title/Abstract]) OR Aseptic Necrosis[Title/Abstract]) OR Ischemic Necrosis[Title/Abstract]) OR Avascular Necrosis[Title/Abstract])
Google Scholar	allintitle:(epiphysis OR epiphyses OR SCFE OR Epiphysiolysis OR SLIPPED OR Vara) (necrosis OR necroses OR osteonecrosis))
Scopus	TITLE (epiphysis OR epiphyses OR SCFE OR Epiphysiolysis OR SLIPPED OR Vara) AND TITLE(necrosis OR necroses OR osteonecrosis)
Cochrane database	#1: epiphysis OR epiphyses OR SCFE OR Epiphysiolysis OR SLIPPED OR Vara<br>#2: necrosis OR necroses OR osteonecrosis<br>#3: #1 AND #2

Table S2. Quality assessment of the included studies

No.	Study ID	Questions assessing the cross-sectional studies								Yes (%)
		1	2	3	4	5	6	7	8	
1	Alshryda 2014	Y	Y	N	Y	Y	N	Y	Y	75.0
2	Alves 2012	Y	Y	N	Y	N	N	Y	Y	62.5
3	Bali 2015	Y	Y	N	Y	Y	N	Y	N	62.5
4	Chen 2009	Y	Y	Y	Y	Y	N	Y	Y	87.5
5	Cosma 2016	Y	Y	N	Y	Y	Y	Y	Y	87.5
6	Davis 2017	Y	Y	N	Y	Y	Y	Y	U	75.0
7	Ilharreborde 2016	Y	Y	N	Y	Y	Y	Y	Y	87.5
8	Kitano 2015	Y	Y	N	Y	Y	N	Y	Y	75.0

9	Kohno 2016	Y	Y	N	Y	Y	Y	Y	Y	87.5
10	Lerch 2019	Y	Y	N	Y	N	N	Y	Y	62.5
11	Madan 2013	Y	Y	N	Y	N	N	Y	Y	62.5
12	Massè 2012	Y	Y	N	Y	N	N	Y	U	50.0
13	Mulgrew 2011	Y	Y	N	Y	N	N	Y	Y	62.5
14	Ng 2019	Y	Y	Y	Y	Y	U	Y	Y	87.5
15	Nortje 2019	Y	Y	N	Y	N	N	Y	Y	62.5
16	Persinger 2016	Y	Y	N	Y	Y	Y	Y	N	75.0
17	Phillips 2001	Y	Y	N	Y	N	N	Y	N	50.0
18	Rached 2012	Y	Y	N	Y	N	N	Y	Y	62.5
19	Sankar 2010	Y	Y	Y	Y	Y	Y	Y	Y	100.0
20	Seller 2006	Y	Y	N	Y	N	N	Y	N	50.0
21	Slongo 2010	Y	Y	N	Y	N	N	Y	Y	62.5
22	Ulici 2017	Y	Y	N	Y	N	N	Y	U	50.0
23	Upasani 2014	Y	Y	N	Y	N	N	Y	Y	62.5
24	Vanhegan 2015	Y	Y	N	Y	Y	N	Y	Y	75.0
25	Walton 2015	Y	Y	N	Y	N	N	Y	Y	62.5
26	Zang 2018	Y	Y	N	Y	N	N	Y	Y	62.5

1. Were the criteria for inclusion in the sample clearly defined? 2. Were the study subjects and the setting described in detail? 3. Was the exposure measured in a valid and reliable way? 4. Were objectives, standard criteria used for measurements of the conditions? 5. Were confounding factors identified? 6. Were strategies deal with confounding factors stated? 7. Were the outcomes measured in a valid and reliable way? 8. Was appropriate statistical analysis used? Y=Yes; N=No; U=Unclear

Table S3. Quality assessment of the included cohort studies

**No.**	**Study ID**	**Questions assessing the cohort studies**											**Yes (%)**
		**1**	**2**	**3**	**4**	**5**	**6**	**7**	**8**	**9**	**10**	**11**	

1	Herngren 2018	Y	N	N	Y	N	Y	Y	Y	Y	N	Y	63.0
2	Jackson 2016	N	N	N	Y	Y	Y	Y	Y	Y	NA	N	60.0
3	Javier 2017	N	N	N	N	N	Y	Y	Y	Y	NA	Y	50.0
4	Lang 2019	N	N	N	N	N	Y	Y	Y	Y	Y	Y	54.0
5	Palocaren 2010	N	N	Y	Y	N	N	Y	Y	Y	NA	Y	60.0
6	Parsch 2009	N	N	Y	N	N	N	Y	Y	Y	NA	N	40.0
7	Souder 2014	Y	Y	Y	Y	Y	Y	Y	Y	Y	NA	Y	100.0

1. Were the criteria for inclusion in the sample clearly defined? 2. Were the studies subjects and the setting described in detail? 3. Was the exposure measured in a valid and reliable way? 4. Were objectives, standard criteria used for measurement of the condition? 5. Were confounding factors identified? 6. Were strategies to deal with confounding factors stated? 7. Were the outcomes measured in a valid and reliable way? 8. Was appropriate statistical analysis used? Y=Yes; N=No; U=Unclear.

(A)

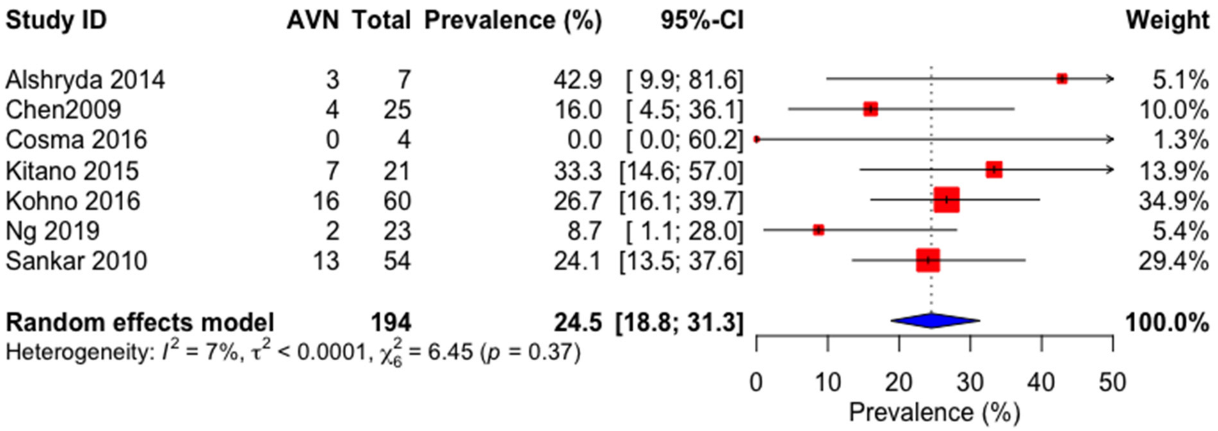

(B)

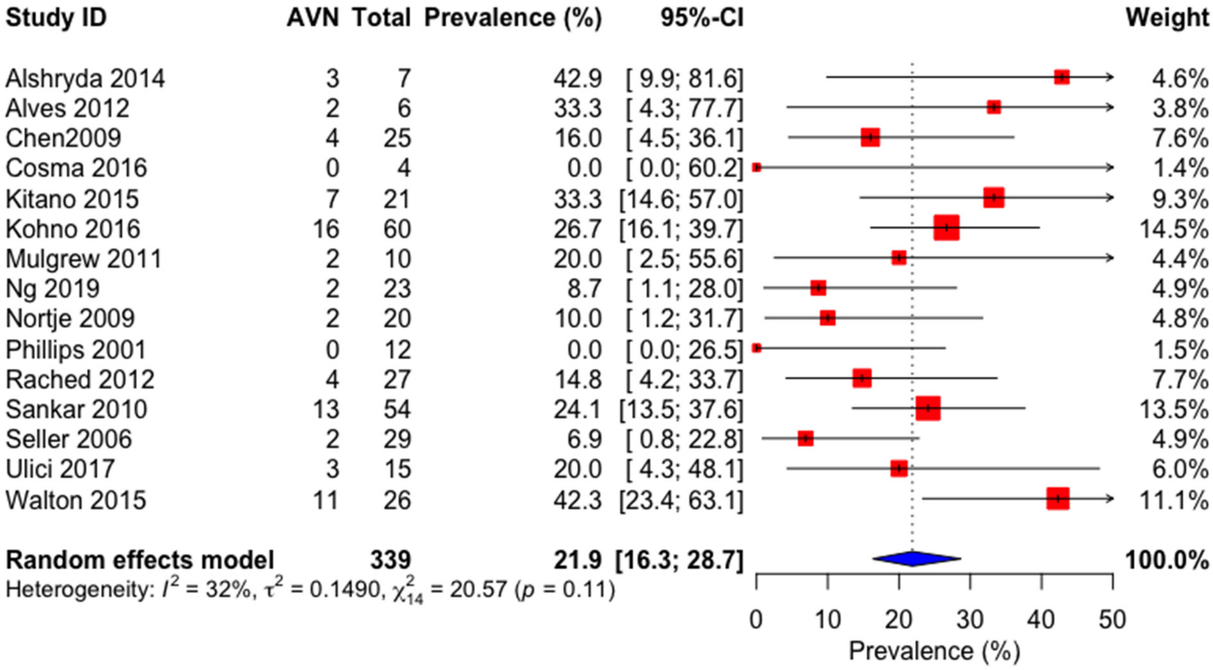
Study ID AVN Total Prevalence (%) 95%-CI Weight
Alshryda 2014 3 7 42.9 [9.9; 81.6] 4.6%
Alves 2012 2 6 33.3 [4.3; 77.7] 3.8%
Chen2009 4 25 16.0 [4.5; 36.1] 7.6%
Cosma 2016 0 4 0.0 [0.0; 60.2] 1.4%
Kitano 2015 7 21 33.3 [14.6; 57.0] 9.3%
Kohno 2016 16 60 26.7 [16.1; 39.7] 14.5%
Mulgrew 2011 2 10 20.0 [2.5; 55.6] 4.4%
Ng 2019 2 23 8.7 [1.1; 28.0] 4.9%
Nortje 2009 2 20 10.0 [1.2; 31.7] 4.8%
Phillips 2001 0 12 0.0 [0.0; 26.5] 1.5%
Rached 2012 4 27 14.8 [4.2; 33.7] 7.7%
Sankar 2010 13 54 24.1 [13.5; 37.6] 13.5%
Seller 2006 2 29 6.9 [0.8; 22.8] 4.9%
Ulici 2017 3 15 20.0 [4.3; 48.1] 6.0%
Walton 2015 11 26 42.3 [23.4; 63.1] 11.1%
Random effects model 339 21.9 [16.3; 28.7] 100.0%
Heterogeneity: I^2 = 32%, τ^2 = 0.1490, χ^2_{14} = 20.57 (p = 0.11)
0 10 20 30 40 50
Prevalence (%)

(C)

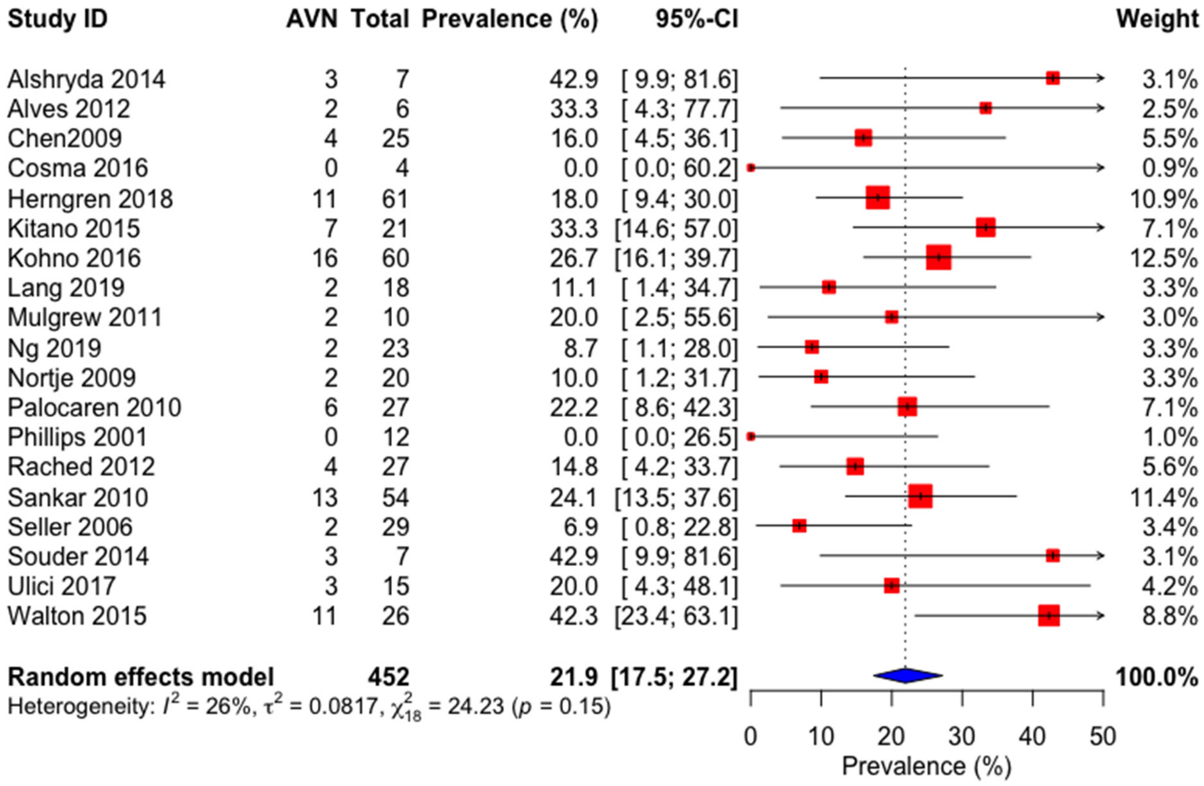
Study ID AVN Total Prevalence (%) 95%-CI Weight
Alshryda 2014 3 7 42.9 [9.9; 81.6] 3.1%
Alves 2012 2 6 33.3 [4.3; 77.7] 2.5%
Chen2009 4 25 16.0 [4.5; 36.1] 5.5%
Cosma 2016 0 4 0.0 [0.0; 60.2] 0.9%
Herngren 2018 11 61 18.0 [9.4; 30.0] 10.9%
Kitano 2015 7 21 33.3 [14.6; 57.0] 7.1%
Kohno 2016 16 60 26.7 [16.1; 39.7] 12.5%
Lang 2019 2 18 11.1 [1.4; 34.7] 3.3%
Mulgrew 2011 2 10 20.0 [2.5; 55.6] 3.0%
Ng 2019 2 23 8.7 [1.1; 28.0] 3.3%
Nortje 2009 2 20 10.0 [1.2; 31.7] 3.3%
Palocaren 2010 6 27 22.2 [8.6; 42.3] 7.1%
Phillips 2001 0 12 0.0 [0.0; 26.5] 1.0%
Rached 2012 4 27 14.8 [4.2; 33.7] 5.6%
Sankar 2010 13 54 24.1 [13.5; 37.6] 11.4%
Seller 2006 2 29 6.9 [0.8; 22.8] 3.4%
Souder 2014 3 7 42.9 [9.9; 81.6] 3.1%
Ulici 2017 3 15 20.0 [4.3; 48.1] 4.2%
Walton 2015 11 26 42.3 [23.4; 63.1] 8.8%
Random effects model 452 21.9 [17.5; 27.2] 100.0%
Heterogeneity: I^2 = 26%, τ^2 = 0.0817, χ^2_{18} = 24.23 (p = 0.15)
0 10 20 30 40 50
Prevalence (%)

(D)

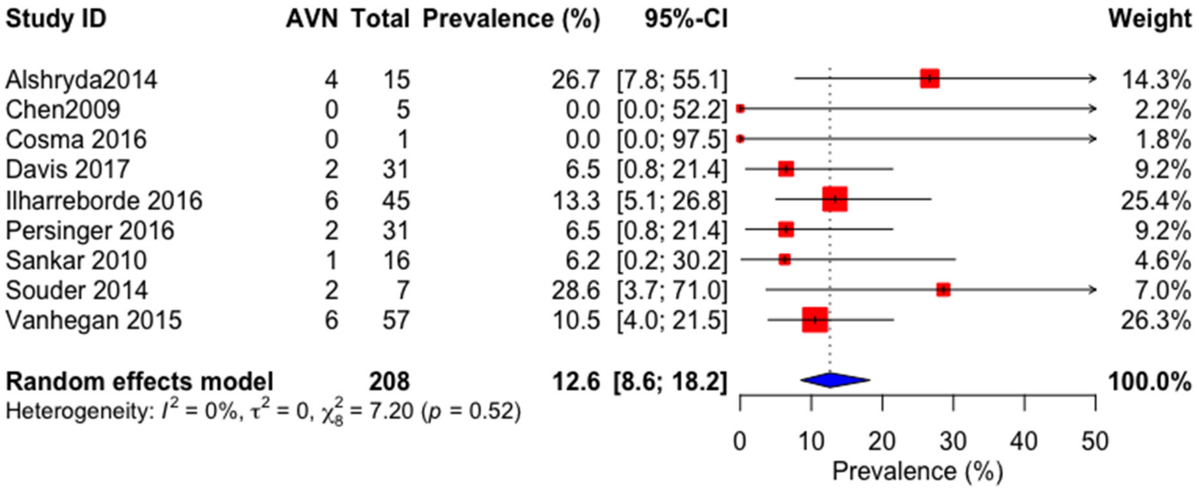

Study ID AVN Total Prevalence (%) 95%-CI Weight
Alshryda2014 4 15 26.7 [7.8; 55.1] 14.3%
Chen2009 0 5 0.0 [0.0; 52.2] 2.2%
Cosma 2016 0 1 0.0 [0.0; 97.5] 1.8%
Davis 2017 2 31 6.5 [0.8; 21.4] 9.2%
Ilharreborde 2016 6 45 13.3 [5.1; 26.8] 25.4%
Persinger 2016 2 31 6.5 [0.8; 21.4] 9.2%
Sankar 2010 1 16 6.2 [0.2; 30.2] 4.6%
Souder 2014 2 7 28.6 [3.7; 71.0] 7.0%
Vanhegan 2015 6 57 10.5 [4.0; 21.5] 26.3%
Random effects model 208 12.6 [8.6; 18.2] 100.0%
Heterogeneity: I^2 = 0%, τ^2 = 0, χ^2_8 = 7.20 (p = 0.52)
0 10 20 30 40 50
Prevalence (%)

(E)

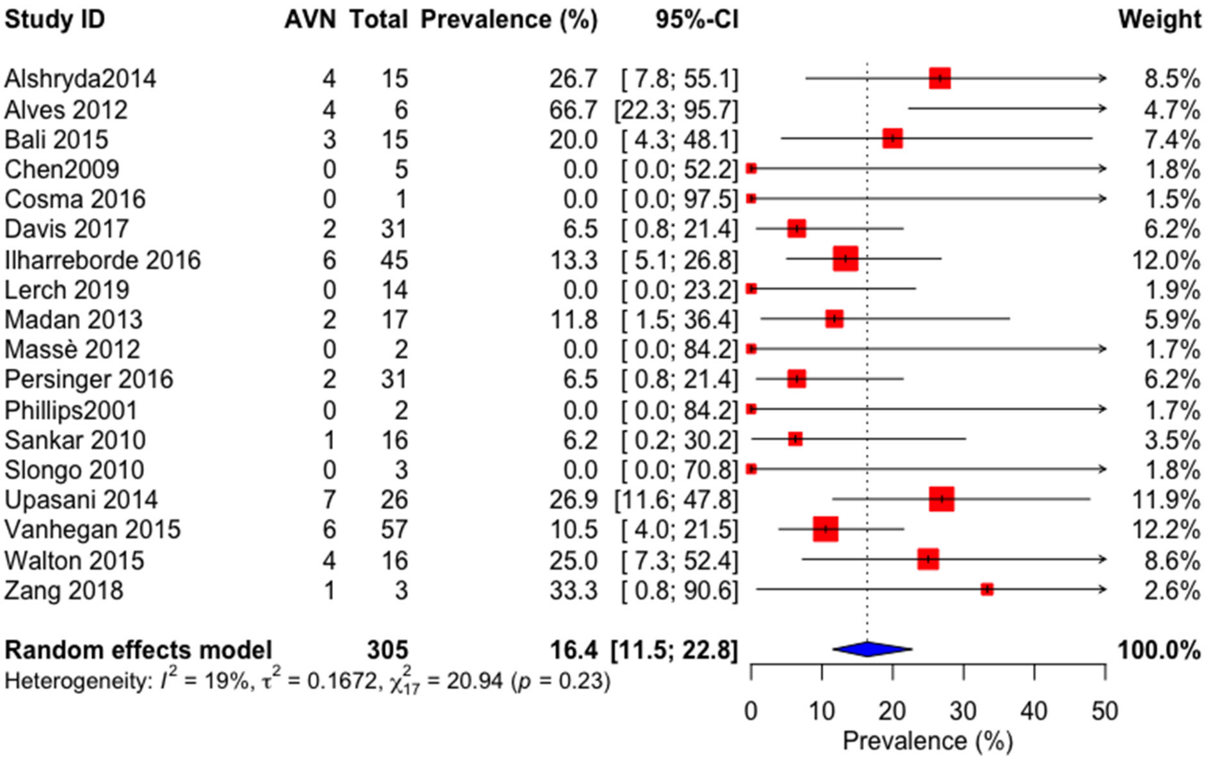

Study ID AVN Total Prevalence (%) 95%-CI Weight
Alshryda2014 4 15 26.7 [7.8; 55.1] 8.5%
Alves 2012 4 6 66.7 [22.3; 95.7] 4.7%
Bali 2015 3 15 20.0 [4.3; 48.1] 7.4%
Chen2009 0 5 0.0 [0.0; 52.2] 1.8%
Cosma 2016 0 1 0.0 [0.0; 97.5] 1.5%
Davis 2017 2 31 6.5 [0.8; 21.4] 6.2%
Ilharreborde 2016 6 45 13.3 [5.1; 26.8] 12.0%
Lerch 2019 0 14 0.0 [0.0; 23.2] 1.9%
Madan 2013 2 17 11.8 [1.5; 36.4] 5.9%
Massè 2012 0 2 0.0 [0.0; 84.2] 1.7%
Persinger 2016 2 31 6.5 [0.8; 21.4] 6.2%
Phillips2001 0 2 0.0 [0.0; 84.2] 1.7%
Sankar 2010 1 16 6.2 [0.2; 30.2] 3.5%
Slongo 2010 0 3 0.0 [0.0; 70.8] 1.8%
Upasani 2014 7 26 26.9 [11.6; 47.8] 11.9%
Vanhegan 2015 6 57 10.5 [4.0; 21.5] 12.2%
Walton 2015 4 16 25.0 [7.3; 52.4] 8.6%
Zang 2018 1 3 33.3 [0.8; 90.6] 2.6%
Random effects model 305 16.4 [11.5; 22.8] 100.0%
Heterogeneity: I^2 = 19%, τ^2 = 0.1672, χ^2_{17} = 20.94 (p = 0.23)
0 10 20 30 40 50
Prevalence (%)

(F)

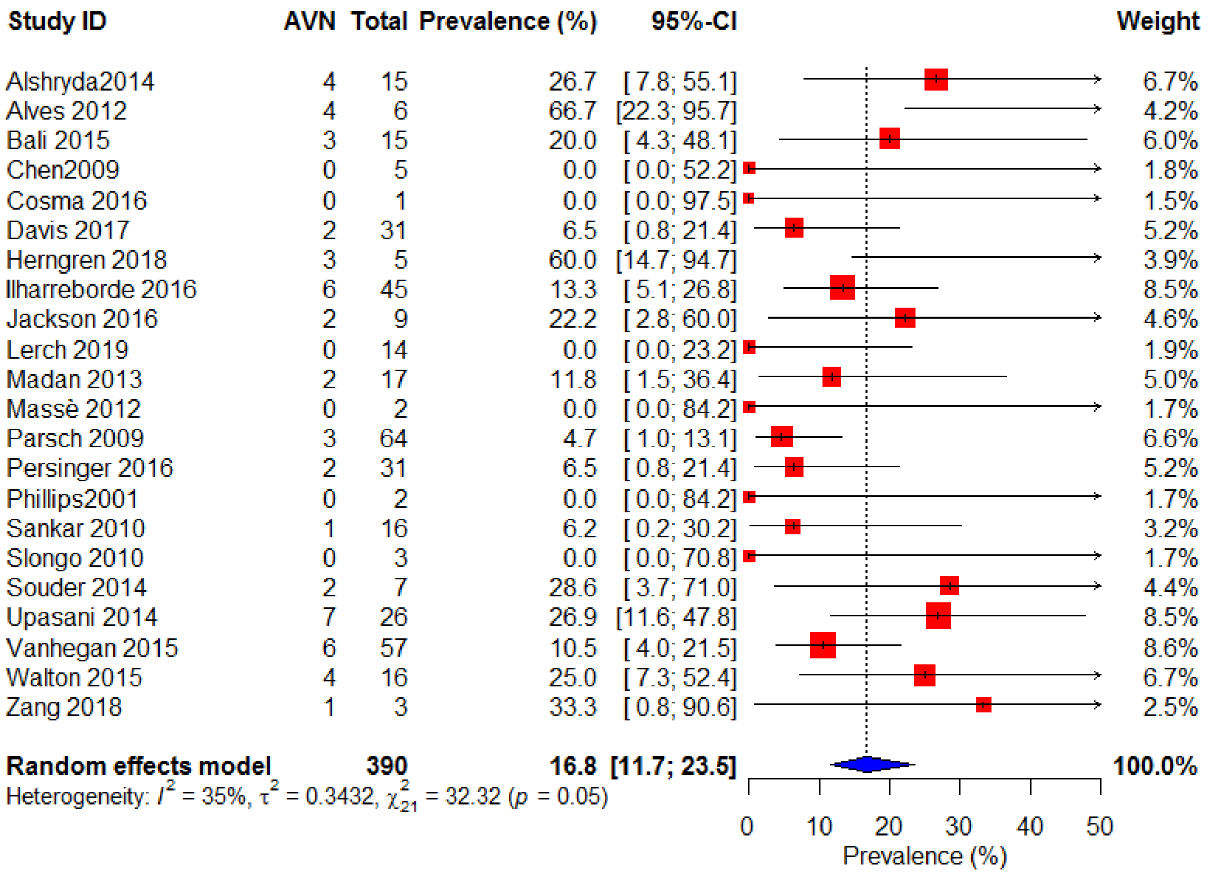

Figure S1. Sensitivity analyses. **(A):** Closed pinning excluding low and moderate quality studies; **(B):** Closed pinning considering only cross sectional studies; **(C):** Closed pinning excluding outlier studies; **(D):** Open reduction excluding low and moderate quality studies; **(E):** Open reduction considering only cross sectional studies; **(F):** Open reduction excluding outlier studies.

(A)

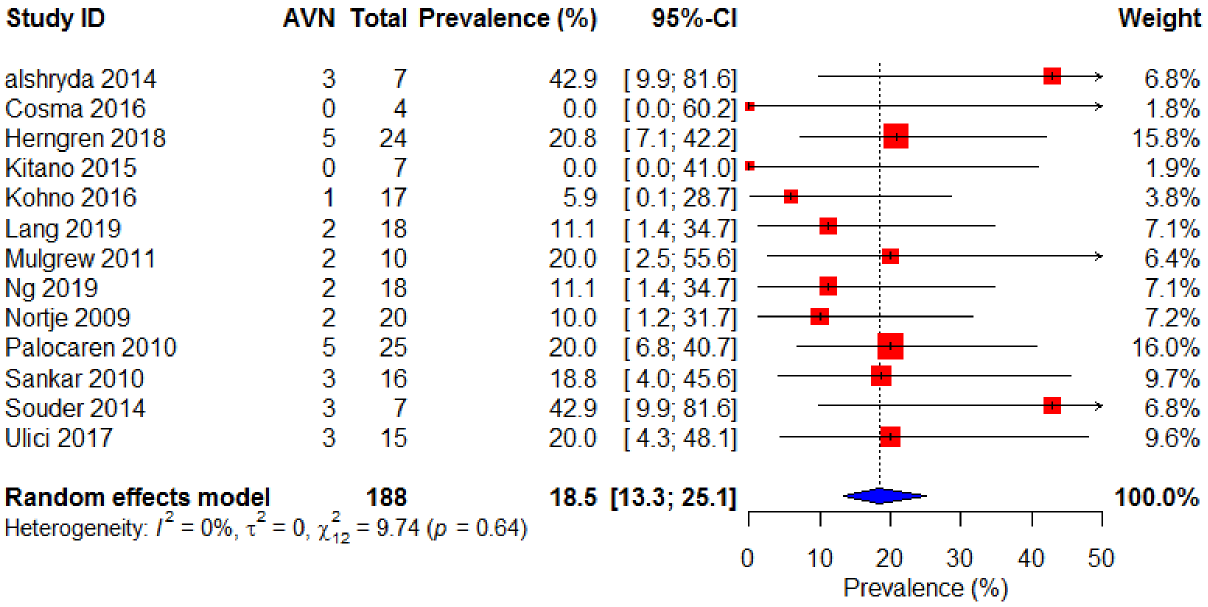

(B)

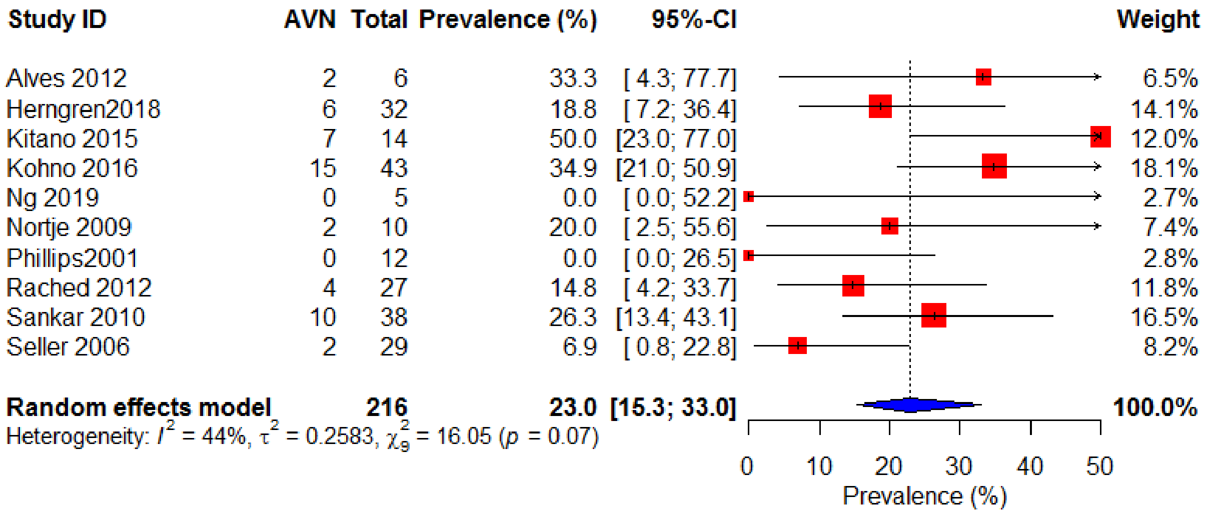
Study ID AVN Total Prevalence (%) 95%-CI Weight
Alves 2012 2 6 33.3 [4.3; 77.7] 6.5%
Herngren2018 6 32 18.8 [7.2; 36.4] 14.1%
Kitano 2015 7 14 50.0 [23.0; 77.0] 12.0%
Kohno 2016 15 43 34.9 [21.0; 50.9] 18.1%
Ng 2019 0 5 0.0 [0.0; 52.2] 2.7%
Nortje 2009 2 10 20.0 [2.5; 55.6] 7.4%
Phillips2001 0 12 0.0 [0.0; 26.5] 2.8%
Rached 2012 4 27 14.8 [4.2; 33.7] 11.8%
Sankar 2010 10 38 26.3 [13.4; 43.1] 16.5%
Seller 2006 2 29 6.9 [0.8; 22.8] 8.2%
Random effects model 216 23.0 [15.3; 33.0] 100.0%
Heterogeneity: I^2 = 44%, τ^2 = 0.2583, χ^2_9 = 16.05 (p = 0.07)
0 10 20 30 40 50
Prevalence (%)

(C)

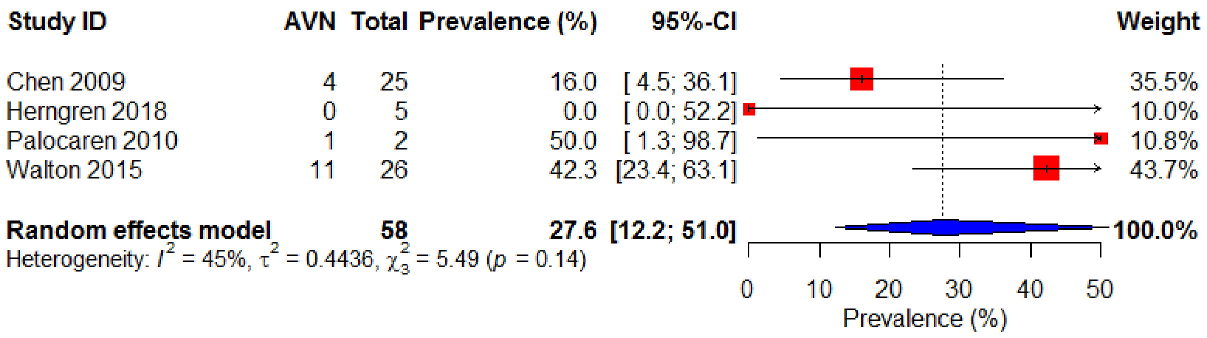
Study ID AVN Total Prevalence (%) 95%-CI Weight
Chen 2009 4 25 16.0 [4.5; 36.1] 35.5%
Herngren 2018 0 5 0.0 [0.0; 52.2] 10.0%
Palocaren 2010 1 2 50.0 [1.3; 98.7] 10.8%
Walton 2015 11 26 42.3 [23.4; 63.1] 43.7%
Random effects model 58 27.6 [12.2; 51.0] 100.0%
Heterogeneity: I^2 = 45%, τ^2 = 0.4436, χ^2_3 = 5.49 (p = 0.14)
0 10 20 30 40 50
Prevalence (%)

(D)

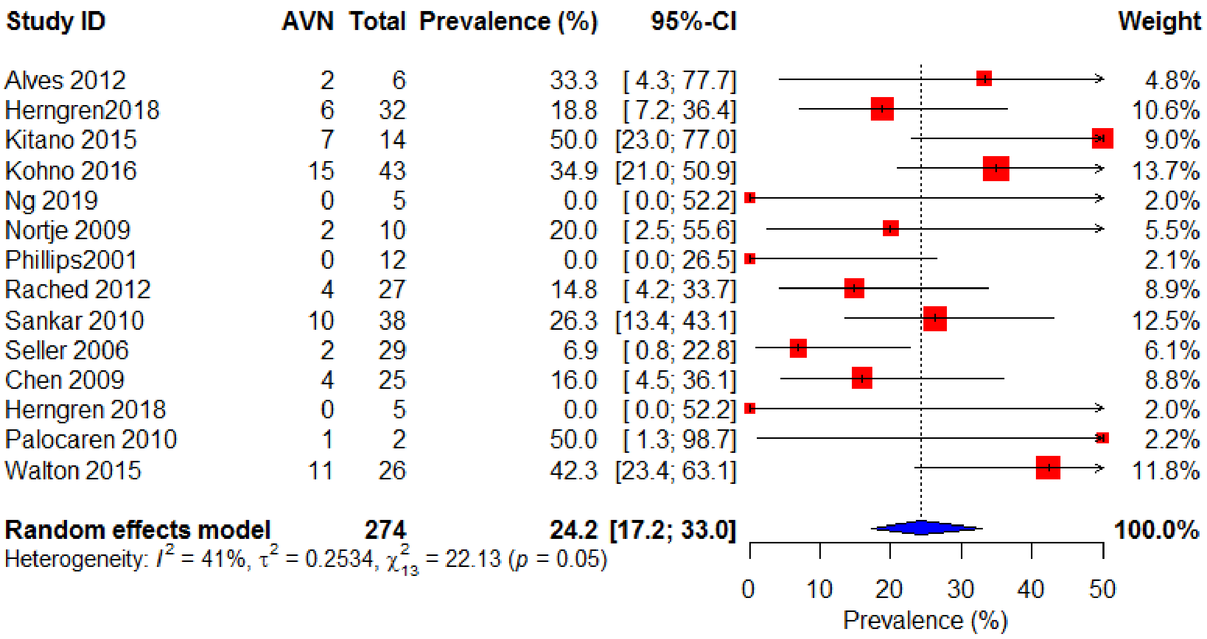
Study ID AVN Total Prevalence (%) 95%-CI Weight
Alves 2012 2 6 33.3 [4.3; 77.7] 4.8%
Herngren2018 6 32 18.8 [7.2; 36.4] 10.6%
Kitano 2015 7 14 50.0 [23.0; 77.0] 9.0%
Kohno 2016 15 43 34.9 [21.0; 50.9] 13.7%
Ng 2019 0 5 0.0 [0.0; 52.2] 2.0%
Nortje 2009 2 10 20.0 [2.5; 55.6] 5.5%
Phillips2001 0 12 0.0 [0.0; 26.5] 2.1%
Rached 2012 4 27 14.8 [4.2; 33.7] 8.9%
Sankar 2010 10 38 26.3 [13.4; 43.1] 12.5%
Seller 2006 2 29 6.9 [0.8; 22.8] 6.1%
Chen 2009 4 25 16.0 [4.5; 36.1] 8.8%
Herngren 2018 0 5 0.0 [0.0; 52.2] 2.0%
Palocaren 2010 1 2 50.0 [1.3; 98.7] 2.2%
Walton 2015 11 26 42.3 [23.4; 63.1] 11.8%
Random effects model 274 24.2 [17.2; 33.0] 100.0%
Heterogeneity: I^2 = 41%, τ^2 = 0.2534, χ^2_{13} = 22.13 (p = 0.05)
0 10 20 30 40 50
Prevalence (%)

(E)

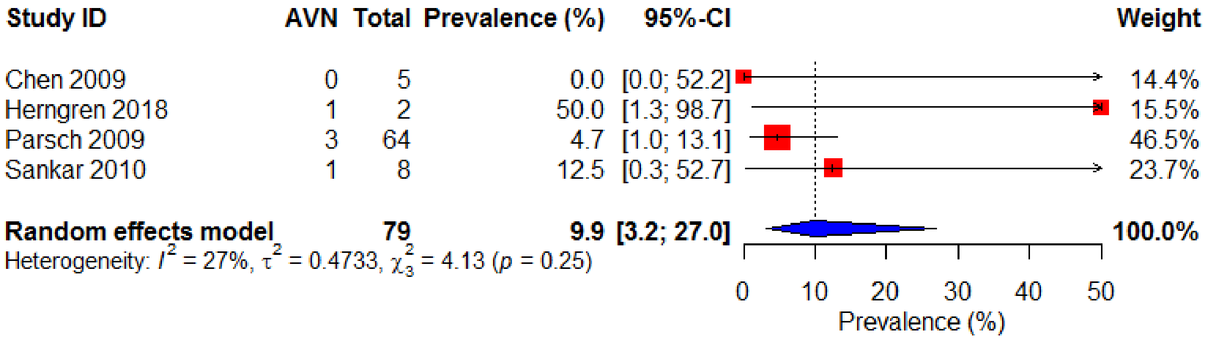

(F)

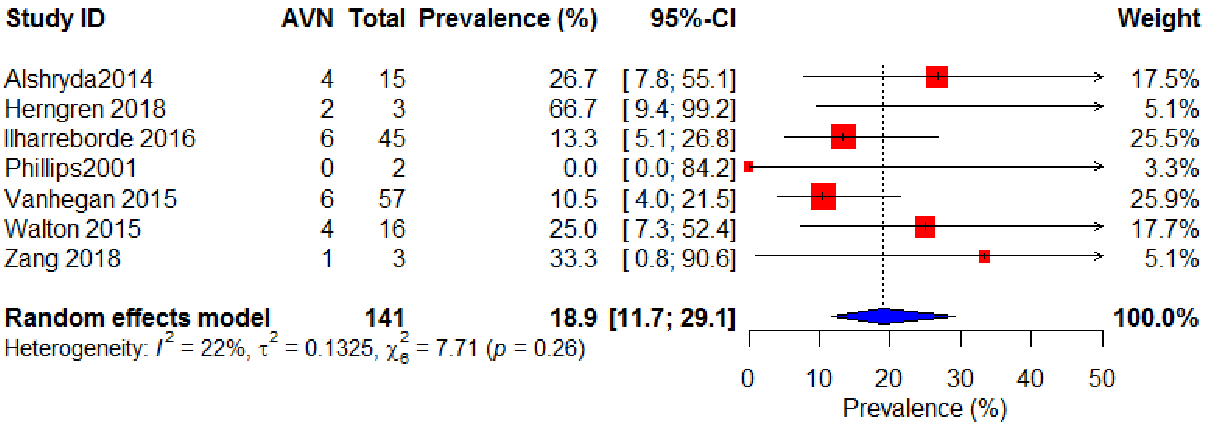

(G)

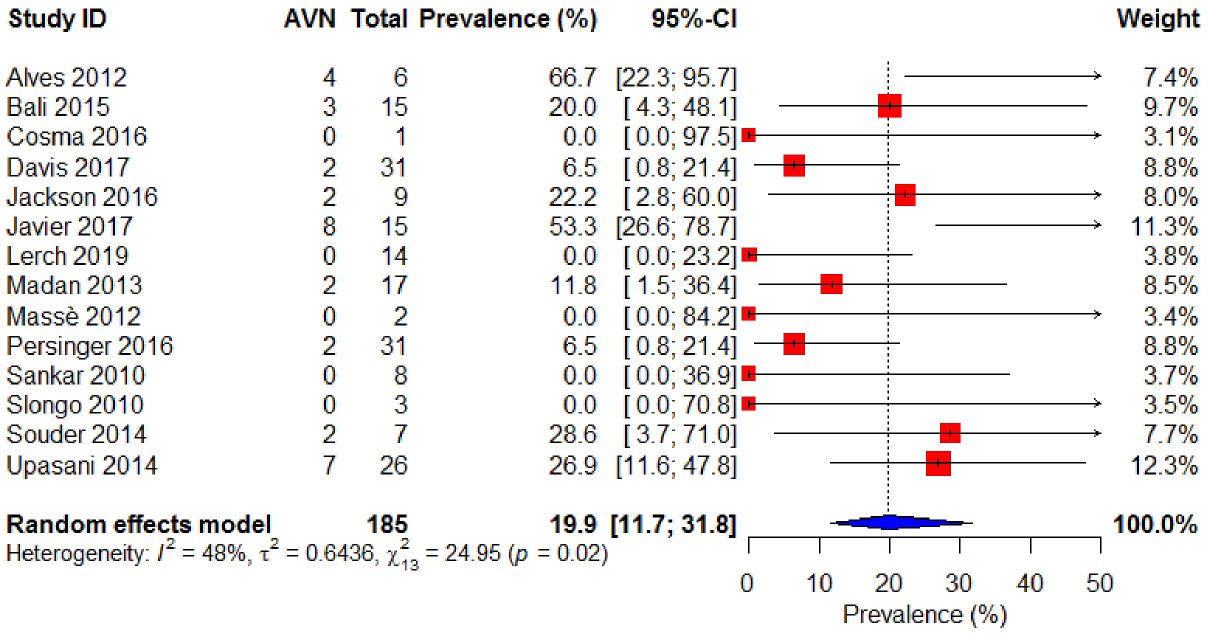

Figure S2. The prevalence of various surgical procedures in slipped capital femoral epiphysis; **(A):** Pinning in situ(PIS); **(B):** Pinning following intentional closed reduction; **(C):** Pinning following unintentional closed reduction; **(D):** Pinning following intentional and unintentional closed reduction; **(E):** Parsch method; **(F):** Subcapital osteotomy(SCO); **(G):** Dunn procedure.
